# Abnormal social reward processing in autism as indexed by pupillary responses to happy faces

**DOI:** 10.1186/1866-1955-4-17

**Published:** 2012-06-07

**Authors:** Leigh Sepeta, Naotsugu Tsuchiya, Mari S Davies, Marian Sigman, Susan Y Bookheimer, Mirella Dapretto

**Affiliations:** 1 Department of Psychology, University of California, Los Angeles, CA, 90095, USA; 2 Division of Humanities and Social Sciences, California Institute of Technology, Pasadena, CA, 91125, USA; 3 Ahmanson-Lovelace Brain Mapping Center, Semel Institute for Neuroscience and Human Behavior, University of California Los Angeles, Los Angeles, CA, 90095, USA; 4 Department of Psychiatry and Biobehavioral Sciences, David Geffen School of Medicine, University of California Los Angeles, Los Angeles, CA, 90095, USA; 5 Children’s National Medical Center, Washington, DC, 20010, USA; 6 School of Psychology and Psychiatry, Monash University, Clayton Campus, Victoria , 3800, Australia; 7 Japan Science and Technology Agency, Tokyo, Japan; 8 Department of Neurology, Child and Adolescent Assessment Division, Sports Concussion Institute Health Services Research group, University of California Los Angeles, Los Angeles, CA, 90095, USA

**Keywords:** Autism, Pupillary response, Reward processing

## Abstract

**Background:**

Individuals with Autism Spectrum Disorders (ASD) typically show impaired eye contact during social interactions. From a young age, they look less at faces than typically developing (TD) children and tend to avoid direct gaze. However, the reason for this behavior remains controversial; ASD children might avoid eye contact because they perceive the eyes as aversive or because they do not find social engagement through mutual gaze rewarding.

**Methods:**

We monitored pupillary diameter as a measure of autonomic response in children with ASD (*n* = 20, mean age = 12.4) and TD controls (*n* = 18, mean age = 13.7) while they looked at faces displaying different emotions. Each face displayed happy, fearful, angry or neutral emotions with the gaze either directed to or averted from the subjects.

**Results:**

Overall, children with ASD and TD controls showed similar pupillary responses; however, they differed significantly in their sensitivity to gaze direction for happy faces. Specifically, pupillary diameter increased among TD children when viewing happy faces with direct gaze as compared to those with averted gaze, whereas children with ASD did not show such sensitivity to gaze direction. We found no group differences in fixation that could explain the differential pupillary responses. There was no effect of gaze direction on pupil diameter for negative affect or neutral faces among either the TD or ASD group.

**Conclusions:**

We interpret the increased pupillary diameter to happy faces with direct gaze in TD children to reflect the intrinsic reward value of a smiling face looking directly at an individual. The lack of this effect in children with ASD is consistent with the hypothesis that individuals with ASD may have reduced sensitivity to the reward value of social stimuli.

## Background

Autism is a pervasive neurodevelopmental disorder characterized by pronounced impairments in social interactions [1]. From the first year of life, individuals with autism spectrum disorders (ASD) show a lack of interest in the human face, and typically show reduced eye contact [2]. The failure to attend to social stimuli such as the human face may have extensive negative consequences for later development. For instance, individuals with ASD may not develop expertise in processing information from faces [3,4] and may fail to appreciate their emotional salience [5].

Why individuals with ASD tend to look away from the eyes and appear to fixate on the local features of the face, such as the mouth and chin, compared to typically developing (TD) counterparts [6-10] remains controversial. One possibility is that they find looking at the eyes over-stimulating or emotionally distressing [10,11]. In support of this “aversion” hypothesis, one study found that in individuals with ASD, eye fixation correlated with activation in the amygdala, an area associated with processing fear [6]. On the other hand, it is also possible that the eyes may simply be uninteresting to individuals with ASD, if they fail to appreciate the information the eye region may convey. According to the social motivation hypothesis, individuals with ASD may not attend to social stimuli—such as faces and the eye region in particular—because they have difficulty forming representations of the reward value of these social stimuli [12,13]. As these individuals may not find faces and eye contact rewarding, they may not be motivated to interact socially. In contrast, a happy face may be socially rewarding for neurotypical individuals. Indeed, happy [14,15] and attractive faces [15,16] have been shown to activate reward circuitry, such as the ventral striatum and orbitofrontal cortex, using functional MRI (fMRI). Further, Kampe et al. (2001) showed that fMRI activation in the ventral striatum is modulated both by reward magnitude (degree of facial attractiveness) and direction of the gaze (direct vs. averted), indicating that reward circuits are engaged when looking at people’s faces and that this network is also sensitive to eye contact. These findings suggest that, for neurotypical individuals, direct eye contact during social interactions may be intrinsically rewarding.

The aim of this study was to investigate whether children and adolescents with ASD find looking at the eye region aversive or simply unrewarding, using pupillometry as an index of emotional responsiveness. Task-evoked pupillary responses have been shown to reflect variations in processing load, or arousal level, across various cognitive domains including short-term memory, language, arithmetic and perception tasks (see [17] for a review). Importantly, emotional arousal, regardless of valence, is also related to the pupillary dilation response [18,19]. In fact, the largest pupillary dilations are evoked by stimuli reported as most aversive or most pleasant [20-22]. Some studies of pupil dilation in children and adolescents have demonstrated greater pupil dilation to negative stimuli (words, interactions) than to neutral or positive stimuli [23,24]. Further, pupillary changes have been found to co-vary with skin conductance change [20], indicating that pupillary responses reflect emotional arousal associated with increased sympathetic activity. Importantly, several studies have reported increases in pupil dilation in response to rewarding stimuli. In adults, sexually arousing stimuli, whether visual or auditory, lead to increases in pupil diameter [25-27]. Increasing levels of reward led to corresponding increases in pupil diameter [28], whether the reward cues were apparent or subliminal. Craving-related visual cues in smokers produced increased pupil diameters [29]; these same cues were also associated with increased fMRI signal in the ventral striatum [30], suggesting that the pupil dilation during craving is related to reward anticipation. However, anticipation of a potential reward or loss in a gambling task caused an increase in pupil diameter, while actually receiving a reward resulted in pupil reduction more than losing a reward, suggesting that pupil dilation may be related to anticipation of reward outcomes rather than the attainment of the reward per se.

Despite the utility of pupillary response as a measure of autonomic arousal, very few studies to date have reported on pupillary response to facial stimuli in developmental disorders, such as autism [32,33]. While one study [32] found overall pupillary constriction when the ASD group viewed faces as compared to the TD group, another study [33] did not show differences in pupillary responses to upright faces between the ASD and TD group. However, this latter study found that the ASD group showed increased pupil dilation to the inverted faces (compared to upright faces), whereas this effect was not seen in the TD group. Overall, little is known about the nature of the pupillary response to faces in individuals with ASD, and the few existing studies have reported discrepant results.

In the current study, we presented emotional faces with averted gaze (the eyes of the faces presented are looking to the side of the subject) and faces with direct gaze (the eyes of the faces presented are gazing directly at the subject). Direct gaze in humans communicates the intent to engage with another person and maintain social interaction [34]. In neurotypical individuals, direct gaze compared to averted gaze is associated with increased activation of the fusiform face area [35]; in contrast, in one study in children with ASD [36], gaze direction was not found to modulate brain activity.

Therefore, in the present study we investigated pupillary response and fixation behavior in children with ASD and age-matched TD controls while they were presented with emotional faces (angry, afraid, happy or neutral) displaying either direct or averted gaze. To examine the aversion hypothesis, we compared pupillary response to emotional faces in the ASD and TD groups. If individuals with ASD avoid looking at faces (and the eye region in particular) because they find this aversive, then children and adolescents with ASD should show an overall increased pupillary response to faces compared to the TD group. However, it is also possible that between-group differences in pupillary responses may only be observed for faces displaying direct eye gaze, irrespective of emotion, or for faces displaying negative affect.

To examine the social motivation hypothesis, we examined pupillary response to faces displaying positive affect (a happy expression) in the ASD and TD groups, with the underlying assumption that a happy face with direct eye gaze would be most rewarding for neurotypical individuals [14,15]. We hypothesized that TD children would show increased pupil dilation to happy faces with direct gaze compared to averted gaze. If individuals with ASD avoid looking at faces, and the eyes in particular, because they find them unrewarding, then our sample of children with ASD should not show this effect.

## Methods

### Ethics statement

This study was approved by the UCLA Office for Protection of Research Subjects, and written informed consent was obtained from every subject.

### Subjects

Subjects were recruited from a pool of subjects previously studied at UCLA, subjects responding to flyers posted around Los Angeles and referrals from the UCLA Autism Clinic. Inclusion criteria for the ASD group were (1) a prior clinical diagnosis of ASD (autism or Asperger’s syndrome) confirmed using the Autism Diagnostic Observation Schedule-Generic [37] and/or the Autism Diagnostic Interview-Revised [38]; (2) no other reported neurological disorders (e.g., cerebral palsy or epilepsy) or structural brain abnormalities; (3) fluent speech and language abilities. TD subjects had no history of medical, psychiatric or neurological disorders according to parental report. Twenty-one (1 female) ASD and 20 (1 female) TD subjects (age 8–18) participated in this study (one ASD and two TD subjects were excluded from the analysis, see below). Verbal, Performance and Full-Scale IQ were assessed by the Wechsler Intelligence Scale for Children-Third Edition (WISC-III) or the Wechsler Abbreviated Scale of Intelligence (WASI). Age and IQ scores (Full-scale, Verbal, Performance) for the two groups are presented in Table 1. No significant differences between the TD and ASD group were observed for age or Performance IQ. Scores on FIQ and VIQ differed between groups; however, “gaze index” for happy faces (see below) was not correlated with FIQ (for ASDs, *r* = −0.305; for TDs, *r* = 0.319; for all, *r* = 0.018) or VIQ (for ASDs, *r* = −0.170; for TDs, *r* = −0.010; for all, *r* = 0.033). None of the correlations were significant (*p* > 0.19).

**Table 1:** Description of subjects

	**ASD group (*n* = 20)**	**TD group (*n* = 18)**
Age (years)	12.4 (2.5)	13.7 (2.7)
FIQ	106 (20)	117 (12)
VIQ	101 (20)	117 (13)
PIQ	110 (18)	112 (11)

Mean and standard deviations of age, Total Verbal, Performance, and Full-Scale IQ for ASDs and TDs.

### Apparatus

**Figure 1:**
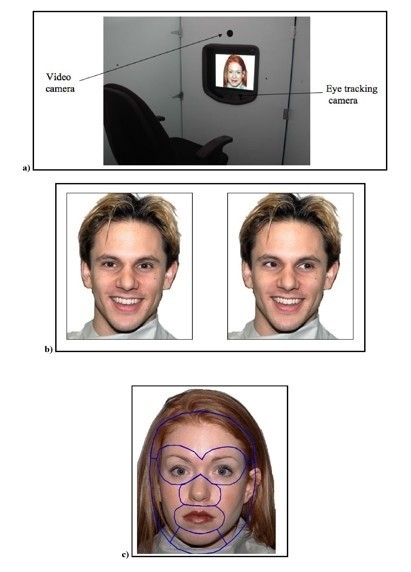
**Study setup, stimuli and regions of interest (ROIs). a** Study setup and apparatus. **b** Example stimuli for the gaze-direct and gaze-averted conditions. **c** Regions of interest around eyes, nose, mouth, chin and forehead, drawn with a 1-cm error margin

Subjects were tested using a video-based eye tracker (Tobii 1750 Eye-Tracking Technology, using ClearView 2.2.0 software, sampling rate of 50 Hz) while viewing static images of emotional faces from the NimStim Face Stimulus Set [39]. During testing, each subject sat 60 cm in front of a monitor equipped with an eye-tracker and viewed the stimuli on the monitor (Figure 1a). The apparatus is able to calculate the x-y coordinates corresponding to the visual fixation points on the screen (frequency of 50 Hz) within 1 cm of accuracy. The monitor was situated within a testing booth designed to minimize visual distractions. The eye-tracking system was calibrated before each session and its accuracy validated before the study.

### Stimuli and procedure

The stimuli and procedure used in this eye-tracking study were the same as those created for a prior fMRI study conducted in our laboratory [36]. We used a subset of the NimStim Face Stimulus Set [39]: 20 young adult individuals, each displaying four emotional expressions (angry, afraid, happy or neutral). Stimuli consisted of an equal number of male and female faces against a white background, with each major racial group represented (Figure 1b). We used the original 80 images for the gaze-direct condition (Figure 1b, left). For the gaze-averted condition, we edited the original 80 images by changing the direction of the pupil and the irises using Adobe Photoshop (Figure 1b, right). Stimulus faces were presented for 2 s, each according to a pseudo-random sequence (ISI = 1 s). To control the initial fixation position, we presented two fixation crosses before each trial. These were presented for 1 s in the same position where the eyes were to appear in the next face stimulus. Null events, consisting of fixation crosses in the center of the screen, were distributed pseudo-randomly throughout the run (as per the prior fMRI protocol). We prepared eight sets of stimulus presentation schedules, which were created by randomizing Gaze Condition (gaze-direct, gaze-averted) and emotional expression. All stimuli presentation schedules were used in each group (approximately an equal number of times). At the beginning of each session, subjects were instructed to pay attention to the pictures and keep their head as still as possible. This was a passive viewing task, and the subject’s level of attention to the stimuli displayed was monitored. One TD subject was unable to follow instructions, and thus his data were excluded from subsequent analysis. Our prior work with this population indicated that high functioning adolescents with ASD have no difficulty identifying and labeling these basic facial emotions [40].

**Figure 2:**
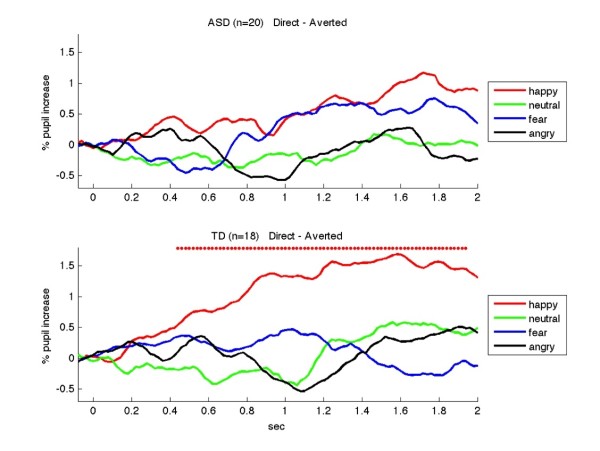
**Differential (i.e., direct-averted) pupillary response time course for TD and ASD groups.** The ASD group is displayed in the *top graph*, and the TD group is shown in the *bottom graph*. TDs showed significantly larger responses to the happy faces (*red*) with direct gaze compared to averted gaze (paired t-test, *p* < 0.05 marked by *circles*)

**Figure 3:**
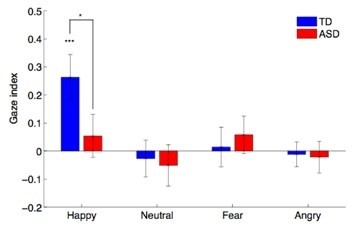
**Gaze index.***Blue*, TDs; *red*, ASDs. Gaze index measures the specificity of the modulation of pupillary response by gaze direction for each emotion. TDs show happy-specific gaze modulation. The *error bars* indicate standard error of the mean

### Pupillary response analysis

Pupil diameter was recorded by the Tobii Eye Tracker. The data were exported and analyzed offline using custom codes in Matlab with Statistics Toolbox. We first removed missing data points (i.e., blinks, fixation outside of the field of view, etc.). Additionally, since pupil diameters are not stable when recorded for less than 100 ms, we removed these unstable points as well. Second, we linearly interpolated the missing data points. Third, the time course was smoothed with a boxcar kernel with a width of 100 ms. The above procedures were independently performed for the pupil data from left and right eyes. Fourth, we extracted the pupillary time course for each trial and rejected trials that contained more than 30% of missing data. If the pupillary time courses were interpolated less than 30% for both eyes, we averaged them. If the data from one eye were rejected, we used the data from the other eye without averaging. At the subject level, we excluded data from subjects who did not have more than 10 trials out of 20 trials in each condition (e.g., direct happy face). By the above procedures, data from one ASD and one TD subject were excluded from all subsequent analyses. For the remaining subjects, 75-100% of trials were valid (for the ASD, on average 93%; for the TD, on average 95%).

For each trial, we converted the pupillary diameter into a percent increase unit, by dividing the time course of the pupillary diameter by the mean pupillary diameter during the 100 ms before the stimulus onset (baseline). We obtained a mean pupillary response time course for each subject by subtracting the mean time course for null trials from the mean time course for all the face trials. We obtained the grand mean pupil response time course across subjects by averaging the mean pupil time course for each subject.

To capture the specificity of the gaze effects on the pupil response to each emotion, we introduced the following gaze index (GI) for emotion, i,

(1) $$GI(i)=\frac{R_{d}(i)-R_{a}(i)}{\sum_{j}|R_{d}(j)-R_{a}(j)|}$$

where R refers to the mean pupillary response between 0.6-1.6 s after stimulus onset; subscripts ‘d’ and ‘a’ refer to directed and averted gaze conditions, respectively. The denominator is the sum of the absolute values of the differential responses over four emotions, j. Gaze index, GI(i), is 1 if and only if emotion, i, elicits a non-zero differential response and the other emotions elicit zero differential responses. If the pupillary response magnitude is the same for all emotions, GI for each emotion is either + .25 or -.25, depending on the polarity of the differential response to direct vs. averted gaze. To compute GI at each time point, we smoothed pupillary response with a boxcar kernel with a width of 200 ms.

### Fixation pattern analysis

Regions of interests (ROIs) were individually drawn around the eyes, mouth, nose, chin and forehead (Figure 1c). Each region had a 1-cm margin to accommodate the resolution of the Tobii Eye Tracker system (~1 degree of visual angle; see Figure 1c). We assigned an ROI value of 1 when the fixation was within a given ROI and 0 otherwise at each sample time (20 ms at 50 Hz). For each subject, we computed the proportion of fixation within a given ROI at each sample time (20 ms) as the probability of fixating on that ROI for each facial stimulus, out of the total number of faces presented (20 faces for each emotion in each gaze condition). Subsequently this proportion was smoothed with a boxcar kernel with a width of 200 ms. To normalize the distribution of the ROI values, we transformed the proportion of fixation with a log-it function for all subsequent statistical analyses;

(2) $$\text{logit}(\text{p})=\frac{1}{2}*\text{log }(\text{p}/(1-\text{p}))\text{.}$$

The logit transformed ROI values were used for the fixation duration analyses.

## Results

### Pupil dilation analyses

#### Analyses to examine the ‘aversion hypothesis’

To examine if individuals with ASD avoid looking at faces, and the eye region in particular, we performed a repeated measure ANOVA with gaze condition (averted and direct) and emotion (happy, angry, fear and neutral) as within-subject factors and diagnosis as a between-group factor (ASD and TD) for pupillary response magnitude, measured as the mean pupillary response from 0.6 to 1.6 s (see Pupillary response analysis section). In this analysis, there was no main effect of group (F(1,36) = 1.399, *p* = 0.245), indicating that the ASD and TD groups displayed similar overall pupillary responses when summing across emotion and gaze condition. Additionally, there was neither a significant group x gaze direction interaction (F(1,36) = 0.399, *p* = 0.532) nor a significant group x emotion interaction (F(3,108) = 1.672, *p* = 0.177), indicating similar pupillary responses across the ASD and TD groups, irrespective of gaze direction and emotions (Table 2). Importantly, we observed no significant main effect of group or group x gaze interaction when we examined pupillary responses to negative emotions (anger and fear) (*p*s > 0.151) or the fear expression alone (*p*s > 0.169). Further, when we examined fear direct vs. neutral direct for the TD and ASD groups the results did not approach statistical significance (TD: F(1,36) = 1.536, *p* = 0.223; ASD: (F(1,36) = 0.158, *p* = 0.694).

**Table 2:** Mean pupillary response for ASD and TD groups

**Emotion**	**Gaze** **condition**	**Group**	**Mean pupil** **increase [%]**	**SD**
Happy	Direct	TD	7.71	2.77
		ASD	8.22	3.77
	Averted	TD	6.37	2.60
		ASD	7.67	4.31
Neutral	Direct	TD	7.10	2.88
		ASD	8.05	4.54
	Averted	TD	7.10	2.79
		ASD	8.21	4.35
Fear	Direct	TD	6.52	3.56
		ASD	8.22	3.88
	Averted	TD	6.33	2.63
		ASD	7.87	4.33
Anger	Direct	TD	6.23	2.82
		ASD	7.86	3.97
	Averted	TD	6.32	2.41
		ASD	8.05	4.57

Mean and standard deviations of pupillary response for each group (ASD and TD), emotion (happy, neutral, fear and anger) and gaze condition (direct and averted).

#### Analyses to examine the ‘social motivation hypothesis’

To examine if individuals with ASD do not look at faces and the eye region in particular because they do not find it rewarding, we examined pupillary responses for the happy emotion using a repeated measure ANOVA with gaze condition (averted and direct) as a within-subject factor and diagnosis as a between-group factor (ASD and TD). This analysis revealed an overall main effect of gaze direction (F(1,36) = 6.446, *p* = 0.016) such that larger pupillary responses were observed for direct gaze across the two groups. While there was no significant main effect of group or a significant group x gaze interaction, as hypothesized, children in the two groups differed in sensitivity to the gaze direction for happy faces. Planned comparisons revealed that the TD group showed a significant effect for gaze direction (F(1,36) = 6.130, *p* = 0.018), with increased pupillary dilation to direct happy faces vs. to averted happy faces. In contrast, the ASD group did not show this effect (F(1,36) = 1.165, *p* = 0.288). Further, in the larger repeated measure ANOVA with both gaze condition and emotion (happy, angry, fear and neutral) entered as within-subject factors, the mean pupillary response for the happy emotion in the direct gaze condition was significantly larger than all of the other emotions in the TD group (F(1,36) = 8.250, *p* = 0.007), but not in the ASD group (F(1,36) = 0.246, *p* = 0.623).

We then performed a second set of analyses where, for each group, the pupillary responses for direct vs. averted gaze were examined for each emotion at each time point. The TD group showed significantly larger pupillary dilation to the gaze-direct happy faces than to the gaze-averted happy faces (Figure 2 top panel, red line; circles indicate *p* < 0.05, for each time point with a paired t-test). For the TD group, this effect was specific to the happy emotion (for all other emotions: *p*s > 0.05 for all time points). The ASD group did not show sensitivity to the gaze direction for any of the emotions (*p*s > 0.05 for all time points, Figure 2 bottom panel).

Finally, to further qualify the effect of gaze direction, we conducted additional analyses using the ‘gaze index,’ which normalizes the differential gaze effects (i.e., gaze direct-gaze averted) by dividing by the sum of the absolute value of the differential gaze effects across all emotions (see Pupillary Response Analysis section in the Methods). A repeated-measure ANOVA with emotion (happy, angry, fear and neutral) as a within-subject factor and diagnosis as a between-group factor (ASD and TD) revealed a main effect of emotion (F(3,108) = 3.825, *p* = 0.012), but no main effect of group (F(1,36) = 1.032, *p* = 0.316) or group x emotion interaction (F(3,108) = 1.210, *p* = 0.310). Importantly, however, the gaze index differed significantly across emotions in the TD (F(3,51) = 4.310, *p* = 0.009), but not the ASD group (F(3,57) = 0.786, *p* = 0.507). A post-hoc non-parametric permutation test (10,000 times [41]) confirmed that the gaze index for happy expressions was larger in the TD group than the ASD group (*p* = 0.038; Figure 3). We conclude that the TD group displayed significantly larger pupillary dilation to happy faces when the gaze was directed to them than when the gaze was averted, whereas the ASD group did not show this effect.

### Fixation pattern analyses

One potential concern is whether the observed pupillary response effects could be secondary to differences in fixation behavior. For instance, the eye region of a face is of high luminance and contrast; increased focus on the eyes in the TD group could result in increased pupil dilation simply due to luminance differences [42]; similarly, faces displaying different emotional expressions could potentially have different luminance properties. Therefore, we examined potential luminance differences across images to rule out this possibility. In each of the eight image categories (four emotions [angry, fear, happy and neutral], two gaze directions [direct vs. averted]), we computed the mean RGB value across the entire image and found no significant differences in luminance (all *p*s > 0.5).

The observed differences in pupillary responses could also be affected by whether attention is directed to the eyes or other parts of the face [10]. It is possible that the TD group might have shown increased pupillary response to the happy faces because they fixated more on the eyes for the direct-gaze than the averted-gaze condition. Similarly, the ASD group might have shown less modulation of the pupillary response by the gaze direction because they fixated less on the eyes than the TD group. Although we attempted to minimize between-group differences by providing fixation crosses at the level of the eyes to cue all participants to look at the eye region, we nevertheless evaluated possible group differences in eye fixation and the relationship between fixation and pupillary responses.

We examined the time that the ASD and TD groups spent fixating on the eyes for each emotion and gaze condition from 0–1.6 s (the time range corresponding to the period from stimulus onset to the end point of the time interval used for the pupillary response analyses). The results of this repeated-measures ANOVA revealed only a significant main effect of emotion (F(3,108) = 6.150, *p* = 0.001) such that participants fixated less on the eyes for the angry than for the neutral faces. Since the significant within- and between-group effects of pupillary response were observed for the happy expressions only, we further probed fixation behavior for this emotion. Overall, the repeated measures ANOVA for happy faces revealed no main effect of group (F(1,36) = 2.347, *p* = 0.134) or gaze condition (F(1,36) = 1.865, *p* = 0.180), or interaction of gaze condition by group (F(1,36) = 0.000, *p* = 0.986) for fixation behavior.

## Discussion

Here we showed increased pupillary responses when TD children and adolescents viewed happy faces with direct vs. averted gaze. In contrast, children and adolescents with ASD did not show any enhanced pupillary response to happy facial expressions (or to any other emotion). Happy faces [14,15] have been shown to activate reward circuitry in neurotypical individuals; in fact, attractive faces displaying direct eye gaze elicit significant activity in the ventral striatum, an area that is part of the neural system associated with reward processing [16]. Numerous studies have also shown that pupillary response may reflect reward processing [19,21,43]. For instance, one reward learning study [21] showed that rewarding stimuli (rated as the “most preferred”) were associated with a significant increase in pupil dilation, as well as increased activity in the ventral striatum. In light of these findings, we suggest that the happy-specific gaze effects of pupillary response observed in our study for the TD group may be related to the intrinsic reward value of a smiling face. The lack of such modulation in the ASD group lends support for the hypothesis that children with ASD have an impairment in social reward processing [13]. Importantly, the present findings are also consistent with recent neuroimaging evidence showing decreased reward circuitry responsivity in children and adolescents with ASD [44].

Previous research has shown that individuals with ASD may display aberrant fixation patterns because they find looking at the eyes aversive [6,11,45]. If this was the case, the ASD group should have exhibited increased pupillary diameters to faces in general, and to faces with direct gaze in particular. However, we did not observe this pattern of results. Further, pupillary responses did not vary in response to a potential threat such as viewing a fearful or an angry face with direct gaze. Taken together, then, our data argue against the ‘aversion hypothesis’ and instead suggest that individuals with ASD may fail to appreciate the reward value of human faces, in line the social motivation hypothesis [13], which posits that individuals with ASD do not find social stimuli and interactions rewarding. However, since other studies have provided data indicating that individuals with ASD may find direct gaze aversive [6,11,45], these hypotheses may not be mutually exclusive. Nonetheless, our study suggests that aversion may not be the only viable account for reduced eye contact seen in autism.

Interestingly, unlike several prior reports (e.g., [23,24,46]), we did not see an overall group difference, or group by gaze interaction, in response to fearful faces. For instance, Farzin, Rivera and Hessl [46] showed greater pupillary response to emotional faces including fear in children with Fragile X syndrome as compared to controls. Both groups showed an increase in pupil size for both happy and fearful expressions. These results are particularly relevant to the current study, as many of the Fragile X subjects in Farzin and colleagues’ [46] study were on the autism spectrum. One possible explanation for the discrepancy between the results of the current study and those observed by Farzin et al. is that our stimuli mainly consisted of faces displaying negative affect (e.g., fear, anger) or neutral facial expressions, which are often evaluated as negative (e.g., [47,48]). Accordingly, the presentation of a happy face may have violated the expectation of a negative facial emotion, leading to a prediction error and thus a larger pupillary response to happy faces in TD children. Indeed, the locus coeruleus, which controls pupil dilation, also plays a role in detecting and responding to new targets in the environment [49,50]. Even 6- to 12-month-old infants show pupil dilation while observing actions that violate their expectations [51]. Lastly, our primary analyses focused on comparisons within emotions but between direct and averted gaze conditions, with an additional comparison of fearful vs. neutral faces with direct gaze. Irrespective of gaze, emotional faces as well as neutral faces may produce similar levels of pupil dilation. For instance, several neuroimaging studies have shown that neutral faces may produce equally strong [52,53] or greater [48] amygdala activation compared to fearful faces, suggesting that neutral faces can be as arousing as those with negative expressions. Thus, pupillary responses in the current study might be predictably smaller than those observed in prior studies that used scrambled faces as control stimuli.

## Conclusions

Typically developing children and adolescents demonstrated increased pupillary diameter to happy faces with direct gaze, suggesting that a smiling face looking directly at an individual is intrinsically rewarding. The absence of this effect in children and adolescents with ASD is consistent with the hypothesis that these children are less motivated to look at faces and make eye contact because of reduced sensitivity to the reward value of these critically important social stimuli. Further studies employing different techniques simultaneously, such as monitoring autonomic responses (pupillary response, skin conductance response and heart rate modulation), tracking eye movements or measuring brain activity may further elucidate the contribution of social motivation deficits to ASD symptomatology.

## Competing interests

The authors declare that they have no competing interests.

## Authors’ contribution

SYB, MD and MSD contributed to the creation of the experimental design. MSD created the stimuli using Adobe Photoshop. LS acquired the data. NT and LS performed the data analyses and jointly wrote the paper. All authors discussed the results, agreed with the interpretation of the findings, and contributed to the writing and editing of the manuscript. All authors read and approved the final manuscript.

## References

[B1] Association AP Diagnostic and statistical manual of mental disorders 1994 4 Washington: Author

[B2] Osterling J Dawson G Early recognition of children with autism: a study of first birthday home videotapes J Autism Dev Disord 1994 24 247 257 10.1007/BF02172225 8050980

[B3] Langdell T Recognition of faces: an approach to the study of autism J Child Psychol Psychiat 1977 19 255 268 681468 10.1111/j.1469-7610.1978.tb00468.x

[B4] Schultz RT Gauthier I Klin A Fulbright RK Anderson AW Volkmar F Skudlarski P Lacadie C Cohen DJ Gore JC Abnormal ventral temporal cortical activity during face discrimination among individuals with autism and Asperger syndrome Arch Gen Psychiatry 2000 57 331 340 10.1001/archpsyc.57.4.331 10768694

[B5] Weeks SJ Hobson RP The salience of facial expression for autistic children J Child Psychol Psychiatry 1987 28 137 151 10.1111/j.1469-7610.1987.tb00658.x 3558531

[B6] Dalton KM Nacewicz BM Johnstone T Schaefer HS Gernsbacher MA Goldsmith HH Alexander AL Davidson RJ Gaze fixation and the neural circuitry of face processing in autism Nat Neurosci 2005 8 519 526 15750588 10.1038/nn1421 PMC4337787

[B7] Klin A Jones W Schultz R Volkmar F Cohen D Visual fixation patterns during viewing of naturalistic social situations as predictors of social competence in individuals with autism Arch Gen Psychiatry 2002 59 809 816 10.1001/archpsyc.59.9.809 12215080

[B8] Neumann D Spezio ML Piven J Adolphs R Looking you in the mouth: abnormal gaze in autism resulting from impaired top-down modulation of visual attention Scan 2006 1 194 202 18985106 10.1093/scan/nsl030 PMC2555425

[B9] Pelphrey KA Sasson NJ Reznick JS Paul G Goldman BD Piven J Visual scanning of faces in autism J Autism Dev Disord 2002 32 249 261 10.1023/A:1016374617369 12199131

[B10] Spezio ML Adolphs R Hurley RS Piven J Analysis of face gaze in autism using “Bubbles” Neuropsychologia 2007 45 144 151 10.1016/j.neuropsychologia.2006.04.027 16824559

[B11] Kylliainen A Hietanen JK Skin conductance responses to another person’s gaze in children with autism J Autism Dev Disord 2006 36 517 525 10.1007/s10803-006-0091-4 16555137

[B12] Dawson G Carver L Meltzoff AN Panagiotides H McPartland J Webb SJ Neural correlates of face and object recognition in young children with autism spectrum disorder, developmental delay, and typical development Child Dev 2002 73 700 717 10.1111/1467-8624.00433 12038546 PMC3651041

[B13] Dawson G Webb SJ McPartland J Understanding the nature of face processing impairment in autism: insights from behavioral and electrophysiological studies Dev Neuropsychol 2005 27 403 424 10.1207/s15326942dn2703_6 15843104

[B14] Phillips ML Bullmore ET Howard R Woodruff PW Wright IC Williams SC Simmons A Andrew C Brammer M David AS Investigation of facial recognition memory and happy and sad facial expression perception: an fMRI study Psychiatry Res 1998 83 127 138 10.1016/S0925-4927(98)00036-5 9849722

[B15] O’Doherty J Winston J Critchley H Perrett D Burt DM Dolan RJ Beauty in a smile: the role of medial orbitofrontal cortex in facial attractiveness Neuropsychologia 2003 41 147 155 10.1016/S0028-3932(02)00145-8 12459213

[B16] Kampe KK Frith CD Dolan RJ Frith U Reward value of attractiveness and gaze Nature 2001 413 589 10.1038/35098149 11595937

[B17] Beatty J Task-evoked pupillary responses, processing load, and the structure of processing resources Psychol Bull 1982 91 276 292 7071262

[B18] Hess EH Polt JM Pupil size as related to interest value of visual stimuli Science 1960 132 349 350 10.1126/science.132.3423.349 14401489

[B19] Steinhauer SR Hakerem G The pupillary response in cognitive psychophysiology and Schizophrenia 1992 New York: NY Academy of Science Press 10.1111/j.1749-6632.1992.tb22845.x 1497258

[B20] Bradley MM Miccoli L Escrig MA Lang PJ The pupil as a measure of emotional arousal and autonomic activation Psychophysiology 2008 45 602 607 10.1111/j.1469-8986.2008.00654.x 18282202 PMC3612940

[B21] O’Doherty JP Buchanan TW Seymour B Dolan RJ Predictive neural coding of reward preference involves dissociable responses in human ventral midbrain and ventral striatum Neuron 2006 49 157 166 10.1016/j.neuron.2005.11.014 16387647

[B22] Steinhauer SR Boller F Zubin J Pearlman S Pupillary dilation to emotional visual stimuli revisited Psychophysiology 1983 20 472

[B23] Silk JS Dahl RE Ryan ND Forbes EE Axelson DA Birmaher B Siegle GJ Pupillary reactivity to emotional information in child and adolescent depression: links to clinical and ecological measures Am J Psychiatry 2007 164 1873 1880 10.1176/appi.ajp.2007.06111816 18056243 PMC2561953

[B24] Silk JS Stroud LR Siegle GJ Dahl RE Lee KH Nelson EE Peer acceptance and rejection through the eyes of youth: pupillary, eyetracking and ecological data from the Chatroom Interact task Soc Cogn Affect Neurosci 2012 7 93 105 10.1093/scan/nsr044 21775386 PMC3252631

[B25] Hess EH Seltzer AL Shlien JM Pupil response of hetero- and homosexual males to pictures of men and women: a pilot study J Abnorm Psychol 1965 70 165 168 14297654 10.1037/h0021978

[B26] Garrett JC Harrison DW Kelly PL Pupillometric assessment of arousal to sexual stimuli: novelty effects or preference? Arch Sex Behav 1989 18 191 201 10.1007/BF01543194 2751414

[B27] Dabbs JM Jr Testosterone and pupillary response to auditory sexual stimuli Physiol Behav 1997 62 909 912 10.1016/S0031-9384(97)00268-0 9284516

[B28] Bijleveld E Custers R Aarts H The unconscious eye opener: pupil dilation reveals strategic recruitment of resources upon presentation of subliminal reward cues Psychol Sci 2009 20 1313 1315 10.1111/j.1467-9280.2009.02443.x 19788532

[B29] Chae Y Lee JC Park KM Kang OS Park HJ Lee H Subjective and autonomic responses to smoking-related visual cues J Physiol Sci 2008 58 139 145 10.2170/physiolsci.RP014207 18358080

[B30] Wang Z Faith M Patterson F Tang K Kerrin K Wileyto EP Detre JA Lerman C Neural substrates of abstinence-induced cigarette cravings in chronic smokers J Neurosci 2007 27 14035 14040 10.1523/JNEUROSCI.2966-07.2007 18094242 PMC2153440

[B31] Satterthwaite TD Green L Myerson J Parker J Ramaratnam M Buckner RL Dissociable but inter-related systems of cognitive control and reward during decision making: evidence from pupillometry and event-related fMRI NeuroImage 2007 37 1017 1031 10.1016/j.neuroimage.2007.04.066 17632014

[B32] Anderson CJ Colombo J Jill Shaddy D Visual scanning and pupillary responses in young children with Autism Spectrum Disorder J Clin Exp Neuropsychol 2006 28 1238 1256 10.1080/13803390500376790 16840248

[B33] Falck-Ytter T Face inversion effects in autism: a combined looking time and pupillometric study Autism Res 2008 1 297 306 10.1002/aur.45 19360681

[B34] Senju A Csibra G Gaze following in human infants depends on communicative signals Curr Biol 2008 18 668 671 10.1016/j.cub.2008.03.059 18439827

[B35] George N Driver J Dolan RJ Seen gaze-direction modulates fusiform activity and its coupling with other brain areas during face processing NeuroImage 2001 13 1102 1112 11352615 10.1006/nimg.2001.0769

[B36] Davies MS Dapretto M Sigman M Sepeta L Bookheimer S Neural bases of gaze and emotion processing in children with autism Brain and Behav 2011 1 1 11 10.1002/brb3.6 PMC3217668 22398976

[B37] Lord C Risi S Lambrecht L Cook EH Jr Leventhal BL DiLavore PC Pickles A Rutter M The autism diagnostic observation schedule-generic: a standard measure of social and communication deficits associated with the spectrum of autism J Autism Dev Disord 2000 30 205 223 10.1023/A:1005592401947 11055457

[B38] Lord C Rutter M Le Couteur A Autism diagnostic interview-revised: a revised version of a diagnostic interview for caregivers of individuals with possible pervasive developmental disorders J Autism Dev Disord 1994 24 659 685 10.1007/BF02172145 7814313

[B39] Tottenham N Tanaka J Leon AC McCarry T Nurse M Hare TA Marcus DJ Westerlund A Casey BJ Nelson CA The NimStim set of facial expressions: judgments from untrained research participants Psychiatry Resin press 10.1016/j.psychres.2008.05.006 PMC3474329 19564050

[B40] Wang AT Dapretto M Hariri AR Sigman M Bookheimer SY Neural correlates of facial affect processing in children and adolescents with autism spectrum disorder J Am Acad Child Adolesc Psychiatry 2004 43 481 490 10.1097/00004583-200404000-00015 15187809

[B41] Efron B Tibshirani RJ An Introduction to the Bootstrap 1998 1 Boca Raton: CRC Press

[B42] Barbur JL Learning from the pupil - studies of basic mechanisms and clinical applications 2004 Cambridge: MIT Press

[B43] O’Doherty JP Dayan P Friston K Critchley H Dolan RJ Temporal difference models and reward-related learning in the human brain Neuron 2003 38 329 337 10.1016/S0896-6273(03)00169-7 12718865

[B44] Scott-Van Zeeland AA Dapretto M Ghahremani DG Poldrack RA Bookheimer SY Reward processing in autism Autism Res 2010 3 53 67 20437601 10.1002/aur.122 PMC3076289

[B45] Joseph RM Ehrman K McNally R Keehn B Affective response to eye contact and face recognition ability in children with ASD J Int Neuropsychol Soc 2008 14 947 955 10.1017/S1355617708081344 18954475

[B46] Farzin F Rivera SM Hessl D Brief report: Visual processing of faces in individuals with fragile X syndrome: an eye tracking study J Autism Dev Disord 2009 39 946 952 10.1007/s10803-009-0744-1 19399604 PMC2684976

[B47] Lee BT Seok JH Lee BC Cho SW Yoon BJ Lee KU Chae JH Choi IG Ham BJ Neural correlates of affective processing in response to sad and angry facial stimuli in patients with major depressive disorder Prog Neuropsychopharmacol Biol Psychiatry 2008 32 778 785 10.1016/j.pnpbp.2007.12.009 18207298

[B48] Thomas KM Drevets WC Whalen PJ Eccard CH Dahl RE Ryan ND Casey BJ Amygdala response to facial expressions in children and adults Biol Psychiatry 2001 49 309 316 10.1016/S0006-3223(00)01066-0 11239901

[B49] Dayan P Yu AJ Phasic norepinephrine: a neural interrupt signal for unexpected events Network 2006 17 335 350 10.1080/09548980601004024 17162459

[B50] Bouret S Sara SJ Reward expectation, orientation of attention and locus coeruleus-medial frontal cortex interplay during learning Eur J Neurosci 2004 20 791 802 10.1111/j.1460-9568.2004.03526.x 15255989

[B51] Gredeback G Melinder A Infants’ understanding of everyday social interactions: a dual process account Cognition 2010 114 197 206 10.1016/j.cognition.2009.09.004 19800056

[B52] van der Gaag C Minderaa RB Keysers C The BOLD signal in the amygdala does not differentiate between dynamic facial expressions Soc Cogn Affect Neurosci 2007 2 93 103 10.1093/scan/nsm002 18985128 PMC2555450

[B53] Kesler-West ML Andersen AH Smith CD Avison MJ Davis CE Kryscio RJ Blonder LX Neural substrates of facial emotion processing using fMRI Brain Res Cogn Brain Res 2001 11 213 226 10.1016/S0926-6410(00)00073-2 11275483

